# Survival association of XRCC1 for patients with head and neck squamous cell carcinoma: A systematic review and meta-analysis

**DOI:** 10.3389/fgene.2022.1035910

**Published:** 2023-01-05

**Authors:** Fan Yang, Liuqing Zhou, Jingcai Chen, Yao Luo, Yanjun Wang

**Affiliations:** Department of Otorhinolaryngology, Union Hospital, Tongji Medical College, Huazhong University of Science and Technology, Wuhan, China

**Keywords:** X-ray repair cross-complementing 1, polymorphism, head and neck squamous cell carcinomas, survival, meta-analysis

## Abstract

**Background:** Epidemiologic studies have demonstrated that X-ray repair cross-complementary group 1 (XRCC1) is one of the susceptibility factors in head and neck squamous cell carcinoma (HNSCC) patients. However, its clinical prognostic impact remains controversial. Thus, a meta-analysis was performed to clarify the association between XRCC1 and the survival outcomes in HNSCC patients.

**Methods:** Following the Preferred Reporting Items or Systematic Reviews Meta Analyses (PRISMA) 2020 guidelines, literature searches were systematically performed in PubMed, EMBASE, Web of Science, Wanfang, and Chinese National Knowledge Infrastructure (CNKI) databases with manual retrieval. Hazard ratios (HRs) and 95% confidence intervals (CIs) were collected to estimate the correlation between XRCC1 and the survival outcomes of HNSCC patients.

**Results:** Ten studies including 1995 HNSCC patients who satisfied the inclusion and exclusion criteria were included in this meta-analysis. Pooled analysis indicated that XRCC1 Arg399Gln and XRCC1 high protein expression were significantly correlated with poor overall survival with HR of 1.31 (95% CIs: 1.03-1.66, *p =* 0.027) and 2.32 (95% CIs: 1.55-3.48 *p* = 0.000) in HNSCC patients. In addition, our results demonstrated that XRCC1 was significantly associated with poor progression-free survival (HR = 1.42, 95% CIs: 1.15-1.75, *p* = 0.001) in HNSCC patients.

**Conclusion**This meta-analysis demonstrated that XRCC1 Arg399Gln and XRCC1 high protein expression increase the risk of poor survival for HNSCC patients. XRCC1 is a potential therapeutic target for HNSCC.

## Introduction

Head and neck squamous cell carcinomas (HNSCC), heterogeneous collection of malignancies of the upper aerodigestive tract, salivary glands and thyroid, constitute 90% of cancers that arise in the head and neck (Leemans et al., 2018; Johnson et al., 2020). HNSCC ranks sixth in the most common cancer worldwide, with 890,000 new cases and 450,000 deaths per year (Sung et al., 2021). Although multidisciplinary treatments for HNSCC have great improvement in the past few years. The incidence of HNSCC continues to rise and the overall disease recurrence remains 40%–50%. Epidemiologic studies indicated that tobacco abuse and alcohol consumption are classical etiologic factors for HNSCC (McDermott and Bowles, 2019). Benzo(a)pyrene from tobacco can induce an increase in reactive oxygen species levels that in turn leads to oxidative DNA damage in the epithelial cells of the head and neck region. The DNA repair pathway in human body can generally correct DNA damage caused by endogenous and environmental agents. Defects in the DNA repair system can also lead to genomic instability and cell death and in turn induce tumorigenesis (Jackson and Bartek, 2009).

Two major classes of DNA repair pathways are base excision repair (BER) and single-strand break repair (SSBR). The primary defense mechanism against oxidative DNA damage is BER (Christmann et al., 2003). X-ray repair cross-complementary group 1 (XRCC1) is a DNA repair scaffold that plays a principle role in BER (Beard et al., 2019). XRCC1 gene is approximately 33 kb length, located on the long arm of the 19th chromosome. XRCC1 protein is 69.5 kDa and consists of 17 exons and 633 amino acids (Vasil’eva et al., 2020). The capacity to repair damaged cells with XRCC1 is encoded by polymorphic genes that may modify the expression of encoded proteins. Single-nucleotide polymorphisms (SNPs) are genetic variants that are related to cancer susceptibility (Nissar et al., 2014). The main variant alleles of XRCC1 gene are Arg399Gln (rs25487) and Arg194Trp (rs1799782). Recently, the survival association of XRCC1 SNPs and XRCC1 protein expression have been reported in various human malignant tumors such as thyroid cancer (Liu and Xue, 2020), lung cancer (Schneider et al., 2008), breast cancer (Sanjari Moghaddam et al., 2016), gallbladder cancer (Wu et al., 2020), and hepatocellular carcinoma (Naguib et al., 2020).

Several studies have investigated that XRCC1 gene polymorphisms (Arg399Gln and Arg194Trp) increase the risk of HNSCC (Choudhury et al., 2014; Dutta et al., 2020; Kabzinski et al., 2021) and suggested that high XRCC1 protein expression is associated with poorer survival in patients with HNSCC(Ang et al., 2011). However, some studies indicated that the XRCC1 Arg399Gln polymorphism do not confer a significant risk for HNSCC (Gal et al., 2005; Wu et al., 2014) and revealed that low expression of XRCC1 statistically significant increase the risk of HNSCC (Kumar et al., 2012). Data from current literature are discordance for the association between XRCC1 and HNSCC survival outcomes. Thus, we aim to conduct a meta-analysis to determine the association between XRCC1 gene polymorphisms, protein expression and survival outcomes in HNSCC patients. The present study followed the Preferred Reporting Items or Systematic Reviews Meta Analyses (PRISMA) 2020 guidelines (Page et al., 2021).

## Methods

### Search strategy

We conducted a systematic computerized search in PubMed, Web of Science, EMBASE, Wanfang, and Chinese National Knowledge Infrastructure (CNKI) databases using the following search terms (XRCC1 or X-ray repair cross-complementing 1) and (prognosis OR outcome OR mortality OR survival OR progression OR recurrence) and (head and neck or laryngeal or tonsil or oropharyngeal or oral or oropharynx or nasopharyngeal) and (squamous cell cancer or carcinoma). Articles published between 1992 and 2022 were considered for study inclusion. The latest search was conducted on 1 June 2022.

### Selection criteria

According to the PICOS (patients, intervention, comparison, outcomes, and study design) principles, the inclusion criteria of the meta-analysis were as follows: 1) Population: patients of any age diagnosed with HNSCC; 2) Intervention: expression of XRCC1 in HNSCC was assessed by immunohistochemical (IHC) analysis. The target gene polymorphisms for XRCC1 were Arg399Gln (rs25487) and/or Arg194Trp (rs1799782) and assessed by polymerase chain reaction restriction fragment length polymorphism (PCR-RFLP); 3) Comparison: HNSCC patients without concentration on high XRCC1 expression or without concentration on XRCC1 Arg399Gln (rs25487) and/or Arg194Trp (rs1799782); 4) Outcomes: overall survival (OS) between XRCC1 and HNSCC as a primary outcome. Progression-free survival (PFS) between XRCC1 and HNSCC as a secondary outcome; 5) Study design: Any human-based studies; 6) including hazard ratios (HR) and the 95% confidence interval (CI) directly, or *p* values with Kaplan- Meier survival curves that can be estimated for OS and/or PFS.

The criteria of exclusion included: 1) abstract-only publications, letters, case reports, meta-analyses, comments, conference articles, on-going or unavailable literature; 2) studies with insufficient original data or focused only on odds ratio without HR values. In case of the same population reported by several publications, the latest literature gained the priority.

### Data extraction

Two researchers (Jing-cai Chen and Yao Luo) independently conducted the electronic search. Data were carefully extracted and cross-checked by two independent researchers (Liu-qing Zhou and Fan Yang) to minimize variation. If there were divergences, the senior researcher (Yan-Jun Wang) participated in the progress of data extraction for achieving a consensus. To begin with, we removed duplicate literature. After manual screening titles and abstracts, publications were eligible for full-text perusal. Finally, studies that meet the selection criteria were included in this meta-analysis. The following information was extracted from each included study: the name of the first author, year of publication, country, cancer type, sample size, age, gender, stage, follow-up time, survival outcomes, method, HR. The Newcastle–Ottawa Scale (NOS) was used to assess the quality of the included publications. A star system (maximum is nine stars) of NOS concentrated in three domains: comparability of study groups, selection of participants and ascertainment of outcomes of interest. Studies with NOS ≥6 were high-quality (Stang, 2010). Reporting recommendations for tumor marker prognostic studies (REMARK) were also applied to evaluate study quality in cancer-related meta-analyses (Sauerbrei et al., 2018).

### Statistical analysis

HRs and 95% CIs are effect measures which were obtained directly from the original data in the selected publications or estimated by *p* values from Kaplan- Meier survival curves following Parmer’s methods (Parmar et al., 1998). OS/PFS were evaluated by pooled HRs and 95% CIs (Tierney et al., 2007). HR = 1 indicates a lack of risk association between XRCC1 and HNSCC. HR > 1 indicates a greater risk of death between XRCC1 and HNSCC. HR < 1 indicates a lower risk of death between XRCC1 and HNSCC. The higher the HR value is, the greater the XRCC1 is related to an increased risk of HNSCC.

Heterogeneity was assessed by the Cochran-based Q test and the I^2^ test (Higgins et al., 2003). *p* < 0.1for Q-test was considered statistically significant heterogeneity between-studies. The fixed-effects model was employed for analysis without obvious statistical heterogeneity between studies (*p* > 0.1, I^2^ ≤ 50%). Otherwise, the random-effects model was applied (Bagos, 2013). Moreover, we performed subgroup analysis to explore the potential source of heterogeneity. To evaluate the strength of the association results, sensitivity analysis was carried out by removing one article at a time and re-measuring the pooled HR. If the pooled HRs did not change, it suggested that our results were not originated from any certain study (Cumpston et al., 2019). Publication bias was assessed by Begg’s funnel plots. And the asymmetry of funnel plot was assessed by the method of Egger’s linear regression test (Begg and Berlin, 1989). The asymmetric plot of Begg’s test and the *p*-value of Egger’s test less than 0.05 were considered a significant publication bias. If publication bias exists, “trim and fill” analysis will be used to adjust the effect of publication bias by removing the small studies with the most extreme results (trim) and recalculating the summary effect size at each iteration until the funnel plot becomes symmetric (Duval and Tweedie, 2000). All the statistical tests used in this meta-analysis were performed with Stata version 12.0 (Stata Corporation, College Station, TX, United States).

## Results

### Study selection and characteristics

**FIGURE 1:**
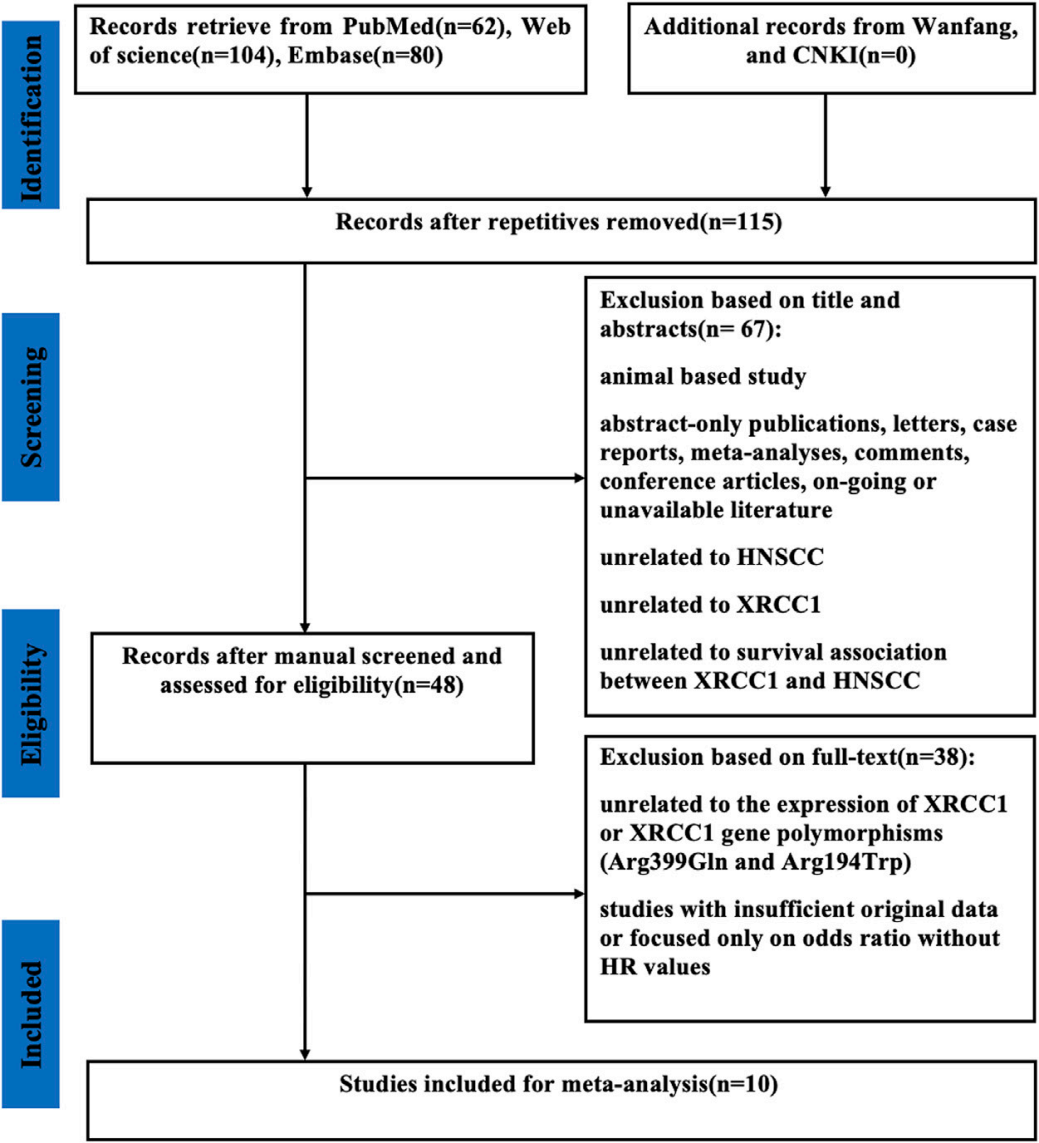
Flow diagram of the selection of relevant studies included in the meta-analysis. CNKI, Chinese National Knowledge Infrastructure; XRCC1, X-ray repair cross-complementary group 1; HNSCC, head and neck squamous cell carcinoma; HR, hazard ratios.

As shown in Figure 1, a total of 246 published articles were selected for initial identification. Of these, 131 duplicate articles were excluded. After screening the full texts of the left 48 publications, we discarded 38 studies due to the lack of insufficient original data or unrelated to the high expression of XRCC1 and XRCC1 gene polymorphisms (Arg399Gln and Arg194Trp). Finally, ten studies with 1995 HNSCC patients were selected in this meta-analysis.

The characteristics of the enrolled studies were summarized in Table 1. Six studies with 1563 patients for HNSCC (Quintela-Fandino et al., 2006; Csejtei et al., 2009; Ang et al., 2011; Azad et al., 2012; Hirakawa et al., 2020; Bold et al., 2021), one study with 134 patients for laryngeal squamous cell carcinoma (LSCC) (Raturi et al., 2020), one study with 98 patients for oral squamous cell carcinomas (OSCC) (Wang et al., 2021), one study with 75 patients for nasopharyngeal carcinoma (NPC) (Jin et al., 2014), and one study with 125 patients for oropharyngeal squamous cell carcinoma (OPSCC) (Costa et al., 2016) were included. Of these, nine studies including 1920 patients reported OS and four studies including 411 patients reported PFS. Sample size of the publications ranged from 75 to 531. Two publications enrolled more than 500 patients. Most of the patients included in this meta-analysis were male and most HNSCC patients were over 45 years old. XRCC1 gene polymorphisms were explored by PCR-RFLP method and IHC method was applied for the expression of XRCC1. Over half of the studies reported the HRs and 95% CIs directly. All the publications’ NOS scores were above 6 and the REMARK scores were between 13 and 16.

**TABLE 1:** Characteristics of the studies included in the meta-analysis. NR, not reported; IHC, Immunohistochemistry; PCR-RFLP, polymerase chain reaction restriction fragment length polymorphism; HNSCC, head and neck squamous cell carcinoma; LSCC, laryngeal squamous cell carcinoma; OSCC, oral squamous cell carcinomas; NPC, nasopharyngeal carcinoma; OPSCC, oropharyngeal squamous cell carcinoma.

Author	Year	Country	Cancer type	Sample size	Age	Gender: Male/female	Stage	Follow-up time	Survival	Method	HR	NOS/REMARK score
Fandino	2006	Canada	HNSCC	103	60.09 (39.09–94.00)	97/6	NR	22.5 (9–51)	OS	PCR-RFLP	Estimated	8/16
Csejtei	2009	Hungary	HNSCC	108	56.7	97/11	I-IV	60	OS	PCR-RFLP	Estimated	6/13
Ang	2011	America	HNSCC	77	56 (39–61)	51/26	I-IV	66 (39–87)	OS, PFS	IHC	Reported	7/14
Azad	2012	Canada	HNSCC	531	63 (33–86)	420/111	I-II	100.52	OS	PCR-RFLP	Reported	7/14
Jin	2014	China	NPC	75	45 (22–72)	57/18	II-IV	25 (5–46)	PFS	PCR-RFLP IHC	Reported	7/14
Costa1	2016	Brazil	OPSCC	125	57 (33–85)	113/12	IV	24.5 (1.5–116.7)	OS, PFS	PCR-RFLP	Reported	7/16
Costa2	2016	Brazil	OPSCC	125	57 (33–85)	113/12	IV	24.5 (1.5–116.7)	OS, PFS	PCR-RFLP	Reported	7/16
Hirakawa	2020	Japan	HNSCC	225	67 (41–91)	200/25	I-IV	48 (3–146)	OS	PCR-RFLP	Estimated	6/14
Raturi	2020	Japan	LSCC	134	56 (32–64)	108/26	III-IV	33	OS, PFS	PCR-RFLP	Reported	6/14
Bold	2021	Germany	HNSCC	519	NR	NR	NR	60	OS	IHC	Estimated	6/13
Wang	2021	China	OSCC	98	51 (31–76)	92/6	I-IV	40 (2.4–137.4)	OS	IHC	Reported	7/15

### Survival association between XRCC1 and HNSCC patients

**FIGURE 2:**
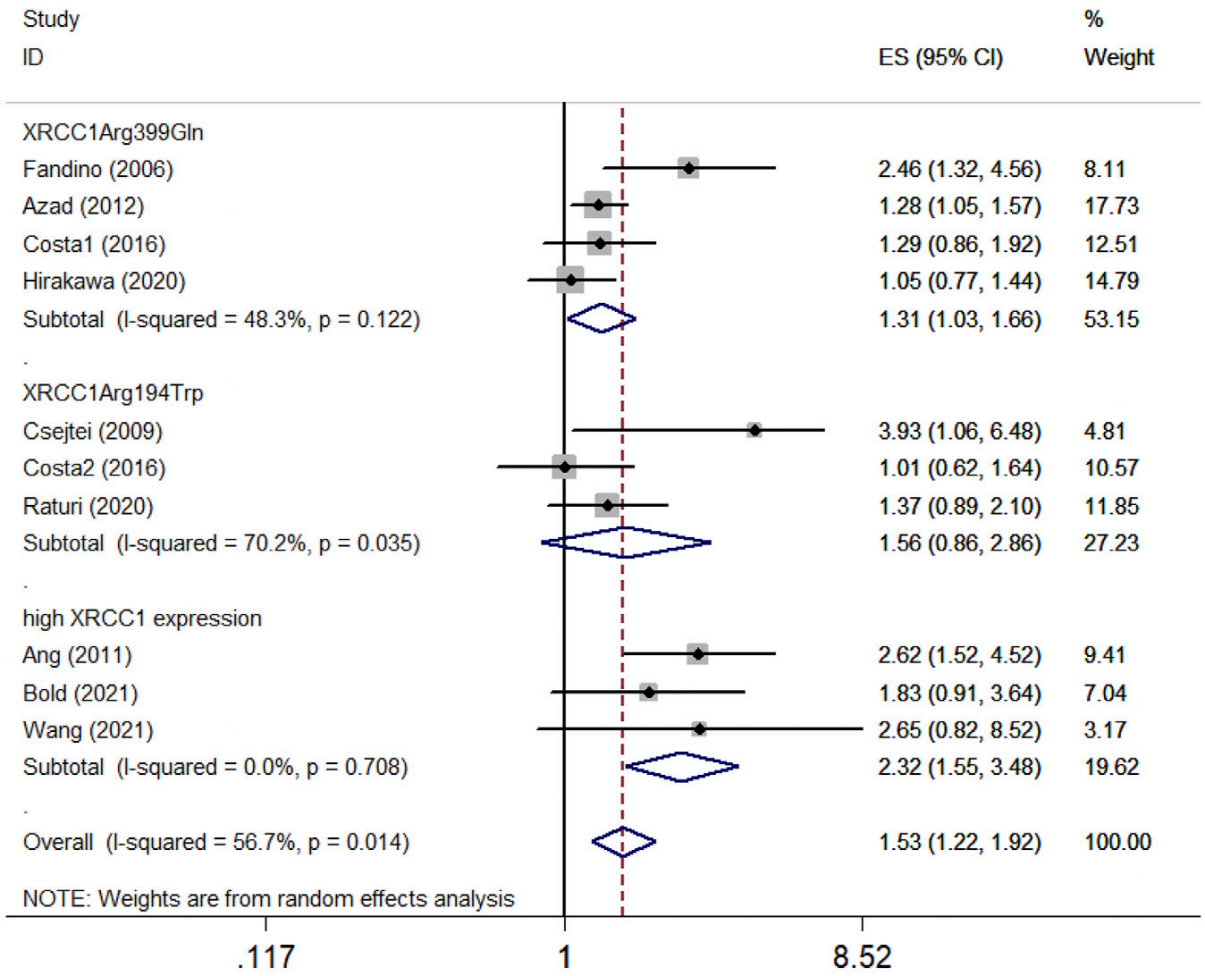
Forest plot indicating the overall survival association between XRCC1 gene polymorphisms (Arg194Trp and Arg399Gln)/high XRCC1 protein expression and HNSCC.

**FIGURE 3:**
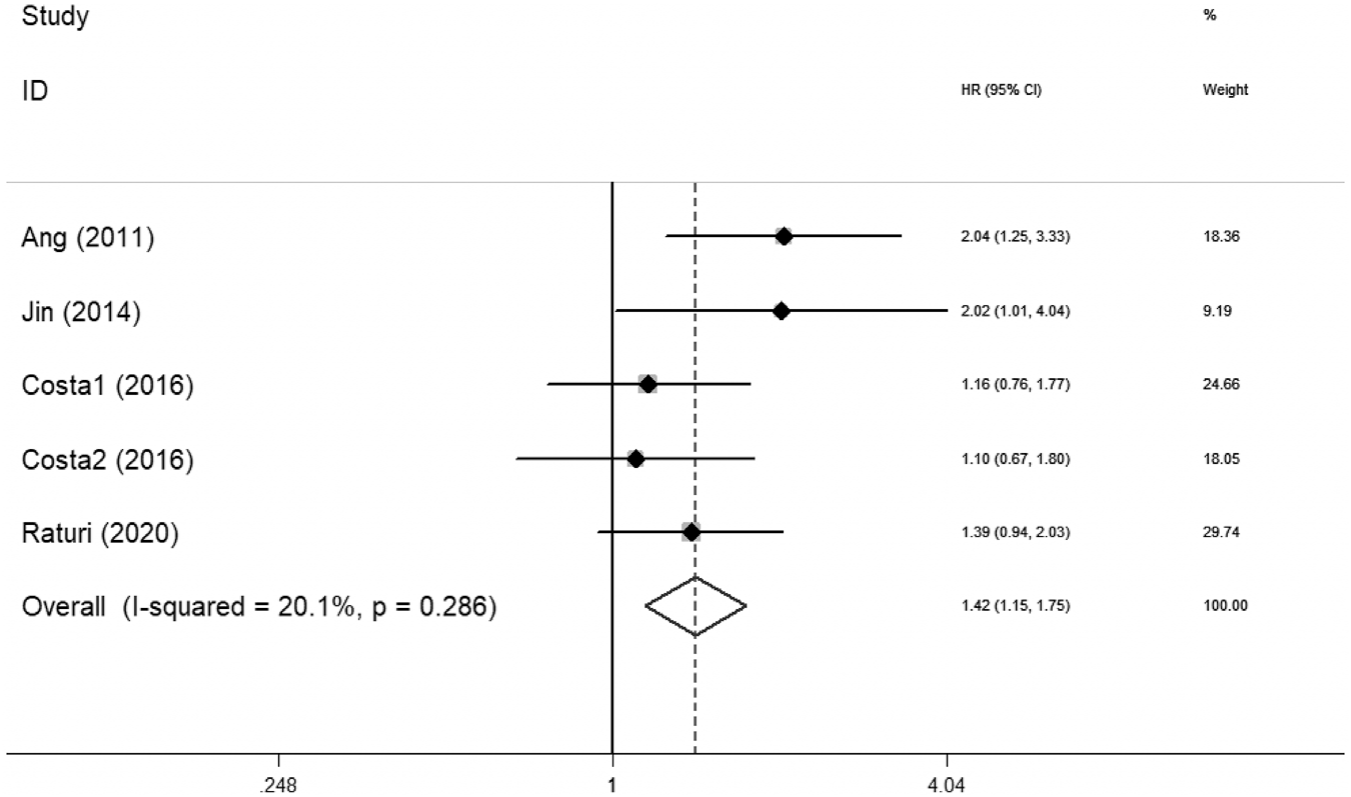
Forest plot indicating the association between XRCC1 and progression-free survival in HNSCC.

Ten articles with 1995 patients included in this meta-analysis evaluated the survival association between XRCC1 and HNSCC. Nine studies including 1920 patients evaluated OS between XRCC1 and HNSCC and four studies including 411 patients evaluated PFS between XRCC1 and HNSCC. The results showed that XRCC1 Arg399Gln (HR = 1.31, 95% CIs: 1.03-1.66, *p* = 0.027) and XRCC1 high protein expression (HR = 2.32, 95% CIs: 1.55-3.48 *p* = 0.000) were significantly correlated with poor OS in HNSCC patients by the random-effects model. However, XRCC1 Arg194Trp was not a risk for HNSCC patients (HR = 1.56, 95% CIs: 0.86-2.86, *p* = 0.146). The degrees of heterogeneity are as follows: I^2^ = 0–25%, no heterogeneity; I^2^ = 25–50%, moderate heterogeneity; I^2^ = 50–75%, large heterogeneity; I^2^ = 75–100%, extreme heterogeneity (Higgins et al., 2003). Moderate heterogeneity was noted between XRCC1 Arg399Gln and OS (I^2^ = 48.3%, *P*_heterogeneity_ = 0.122). Large heterogeneity was noted between XRCC1Arg194Trp and OS (I^2^ = 70.2%, *P*_*heterogeneit*y_ = 0.035). No heterogeneity was noted between high XRCC1 expression and OS (I^2^ = 0.0%, *P*_*heterog*eneity_ = 0.708). Overall, the pooled heterogeneity of XRCC1 in OS was large (I^2^ = 56.7%, *P*_*heterogeneit*y_ = 0.014) (Figure 2). Significant correlation between XRCC1 and poor PFS (HR = 1.42, 95% CIs: 1.15–1.75, *p* = 0.001) was observed with no heterogeneity (I^2^ = 20.1%, *P*_*heterogeneity*_ = 0.286) (Figure 3).

### Sensitivity analysis

**FIGURE 4:**
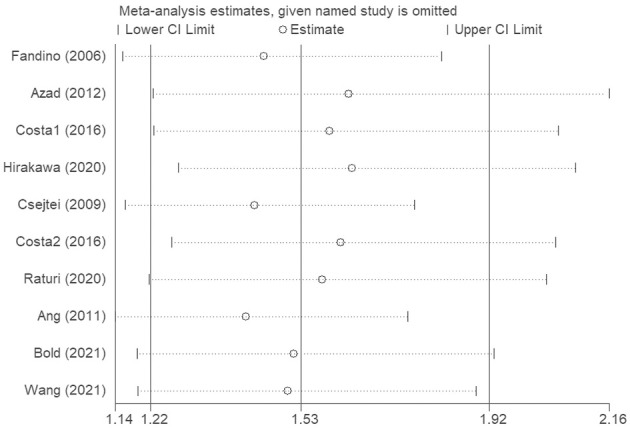
The sensitivity analysis was conducted to evaluate the effects of each single study on the overall effect.

The sensitivity analysis was conducted to evaluate the effects of each single study on the overall effect. We conducted a leave-one-out sensitivity analysis to evaluate the effect of each single data point against the aggregate. The recalculated outcomes (Figure 4) were not substantially influenced, suggesting that the combined effect size of the meta-analysis results was stable and reliable.

### Publication bias

**FIGURE 5:**
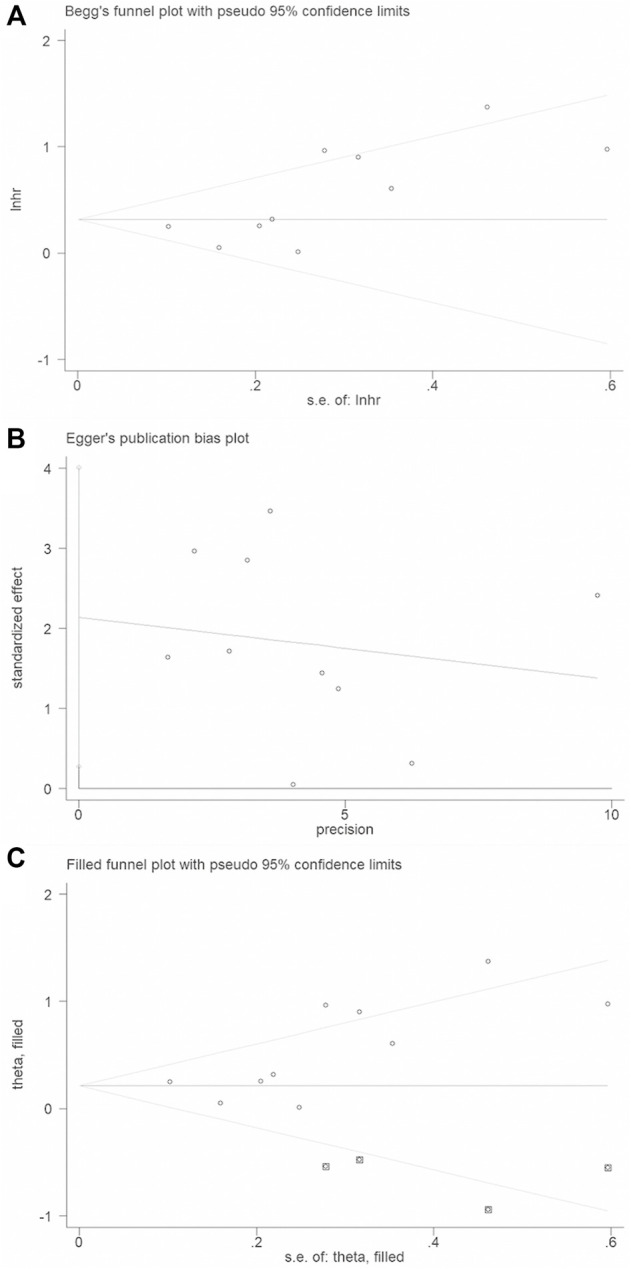
Publication bias and trim and fill analysis of the enrolled analysis: **(A)**. The Begg’s funnel plot; **(B)**. The Egger’s test. **(C)**. Trim and fill analysis.

Begg’s funnel plot and Egger’s test were applied to validate potential bias from searched publications. We observed that two sides of the Begg’s funnel plot were asymmetric. The Egger’s test indicated publication bias existed (*p* = 0.03). Therefore, “trim and fill” analysis was further utilized and the pooled HR was 1.246 (95% CIs: 0.967–1.607) (Figure 5).

## Discussion

In the present meta-analysis, we included ten studies with 1995 HNSCC patients. Results indicated that the XRCC1 gene polymorphism Arg399Gln and XRCC1 high protein expression were significantly associated with poor OS for HNSCC patients and XRCC1 was significantly associated with poor PFS. There was large heterogeneity in our study (I^2^ = 56.7%, *P*_*heterogeneity*_ = 0.014). *p* < 0.10 suggested the heterogeneity is significant and I^2^>50% indicated the heterogeneity is large across studies. The random-effects mode was employed for analysis with large significant heterogeneity across studies (*p <* 0.1, I^2^>50%). The fixed-effects model would be performed for analysis with no obvious heterogeneity between studies (*p >* 0.1, I^*2*^≤50%). Considering the existence of large-significant heterogeneity in our meta-analysis, a random-effects model was chosen for the generation of pooled indexes. The sensitivity analysis confirmed this meta-analysis is stable and reliable. The asymmetric funnel plot and the Egger’s test (*p =* 0.03) conferred that a significant publication bias existed in our study. Thus, we implemented “Trim and fill” analysis to judge the impact of publication bias. The method of “Trim and fill” analysis was conducted by removing the small studies with the most extreme results (trim) and recalculating the summary effect size at each iteration until the funnel plot becomes symmetric. Results showed that the point estimate of the overall effect size is approximately correct in our study (HR = 1.282, 95% CI: 0.98–1.677).

Anti-cancer drug therapy for HNSCC has been widely used in the clinic and has recently been shown to be effective. In a previous study, oncogene genes in HNSCC were identified through extensive DNA sequencing and genetic analysis (2015). Therefore, it is reasonable to quest potential biomarkers of treatment by analyzing genomic features.

DNA repair genes have been considered driver genes of HNSCC due to their frequent mutation. Defects in DNA repair promote genomic instability and carcinogenesis (Tubbs and Nussenzweig, 2017). DNA repair systems play an indispensable role in protecting cells against carcinogenic agents from internal and external stimuli (Leemans et al., 2011). Statistical analyses indicated that the DNA repair status is associated with poor prognosis in cancers (Birkbak et al., 2011; Azad et al., 2012). XRCC1 is a major DNA repair gene involved in BER for small base lesions resulting from oxidation and alkylation damage (Almeida and Sobol, 2007; Lou et al., 2013). More than 300 validated SNPs in the XRCC1 gene were reported in the dbSNP database (http://www.ncbi.nlm.nih.gov/SNP). Among them, the XRCC1 gene polymorphisms (Arg194Trp and Arg399Gln), which occur within conserved sequences, are the most frequently mutated and the most extensively studied.

Studies have investigated the association between XRCC1 Arg399Gln/XRCC1 Arg194Trp polymorphisms and HNSCC risk (Li et al., 2007; Majumder et al., 2007; Applebaum et al., 2009; Gugatschka et al., 2011; Kostrzewska-Poczekaj et al., 2013; Jin et al., 2014; Raturi et al., 2020). However, these results were contradictory. Wang et al. (2013) conducted a meta-analysis on the association of XRCC1 Arg399Gln polymorphisms with HNSCC risk. Nevertheless, they did not observe any precise estimation of this relationship based on 18 published studies. Sturgis (Sturgis et al., 1999) reported a reduced risk between XRCC1 Arg194Trp and HNSCC while Andrew (Olshan et al., 2002) found a weak elevation between HNSCC risk and the XRCC1 Arg194Trp polymorphism. XRCC1 protein is involved in BER (Thompson and West, 2000; Weaver et al., 2005) and its protein expression alters the sensitivity of cells to radiation and chemotherapeutic agents (Park et al., 2002). It was reported that high protein expression levels of XRCC1 may be a risk factor for HNSCC (Ang et al., 2011; Wang et al., 2021). In addition, XRCC1 protein expression is common in HNSCC and high XRCC1 protein expression may confer poorer survival, regardless of the primary tumor site or stage (Ang et al., 2011; Bold et al., 2021). In summary, these results either contrast with each other or are not accurate conclusions.

This meta-analysis prospectively evaluated XRCC1 as a bio-predictor of survival outcomes in patients with HNSCC. There is no doubt that our study is the first meta-analysis including ten published studies with 1995 patients to comprehensively evaluate the survival value of XRCC1 (SNPs and high protein expression) in HNSCC. It might offer useful information for clinical decision-making in HNSCC.

After analyzing and summarizing all selected data, the results indicated that high protein expression and Arg399Gln SNPs of XRCC1 significantly predicted poor OS in HNSCC patients with HRs of 2.32 and 1.31. These findings confirmed that XRCC1 could be widely applied as a diagnostic marker and therapeutic target in HNSCC patients. As a genetic-associated study, the Hardy-Weinberg (HWE) principle was used to avoid methodological weaknesses, such as biased selection of subjects or genotyping errors. The enrolled studies in our meta-analysis were all in agreement with HWE principle.

This meta-analysis should be interpreted within the context of its limitations. First, all included studies are published with English languages only. Therefore, publication bias is very likely to occur. Second, the number of articles was limited and the sample size was relatively small in the present meta-analysis. False-positive or false-negative findings may have occurred in small sample sizes. Therefore, larger scale and comprehensive studies are needed to achieve a more persuasive conclusion. Third, large heterogeneity was observed in this meta-analysis. The heterogeneity of the included studies was likely due to differences of the baseline characteristics in patients or different sites of HNSCC or tumor treatments or HRs calculated by Parmer’s methods or other parameters. A random-effects model was conducted to minimize the effects of these differences. Forth, studies with positive results are more likely to be published and thus more likely to be enrolled. Hence, the survival association of XRCC1 in HNSCC may to some extent has been overestimated in this meta-analysis.

## Conclusion

In conclusion, our meta-analysis demonstrated that XRCC1 gene polymorphism Arg399Gln and high protein expression of XRCC1 were associated with poor OS in HNSCC patients. And XRCC1 was significantly associated with poor PFS in HNSCC patients. This current meta-analysis might provide favorable data for future application of XRCC1 as bio-predictor for HNSCC treatment. Larger prospective studies should be conducted in the future to further verify the results in the present study.
